# Changes in chronic neck pain following the introduction of a visco-elastic polyurethane foam pillow and/or chiropractic treatment

**DOI:** 10.4102/hsag.v24i0.1099

**Published:** 2019-10-09

**Authors:** Laura J. Soal, Charmaine M. Bester, Brandon S. Shaw, Chris Yelverton

**Affiliations:** 1 Department of Chiropractic, University of Johannesburg, Johannesburg, South Africa; 2 Department of Human Movement Science, University of Zululand, KwaZulu-Natal, KwaDlangezwa, South Africa

**Keywords:** cervical pain, cervical pillow, manipulation, manual therapy, mobilisation, sleep

## Abstract

**Background:**

Sleep ergonomics are increasingly prescribed as an adjunct treatment to chronic neck pain. Postulated benefits to maintaining the ideal sleeping posture are improved tissue repair in and around the facet joints, decrease in tension of associated musculature and better quality sleep.

**Aim:**

The purpose of this study was to determine if the inclusion of a visco-elastic polyurethane (VEP) pillow could benefit the chiropractic treatment of chronic neck pain.

**Setting:**

The study took place at a chiropractic training clinic in Johannesburg.

**Method:**

Participants were randomly assigned to either a chiropractic treatment only group (CHI) (*n* = 15) or a chiropractic treatment with a VEP pillow group (CHI+P) (*n* = 15). Both groups underwent six chiropractic treatments spaced at 3–4-day intervals and the CHI+P were provided with a VEP pillow. Baseline and post-test measurements consisted of the initial Numerical Pain Rating Scale (NRS) and the Vernon–Mior Neck Pain and Disability Index (NDI).

**Results:**

Both the CHI and CHI+P groups significantly (*p* ≤ 0.05) improved their NRS (*p* = 0.001 for both groups) and NDI (*p* = 0.001 and *p* = 0.000, respectively) scores. Furthermore, post hoc analysis indicated a significant difference at post-test between the two groups for NRS (*p* = 0.015), but not for NDI (*p* = 0.195). The CHI+P demonstrated an improved minimum clinically important difference (MCID) (43% vs. 73% for NRS and 59% vs. 71% for the NDI).

**Conclusion:**

Findings of this study suggest that a VEP pillow could be included as an adjunct management tool to chiropractic treatment of chronic neck pain.

## Introduction and background

Neck pain is a common health problem with approximately 70% of the population suffering from its debilitating effects at some point in their lives (Bronfort et al. 2001). Neck pain often becomes chronic with a 12-month prevalence ranging from 30% to 50% (Hogg-Johnson, Van der Velde & Carrol 2008). Moreover, worsening pain is associated with poor health-related quality of life (Nolte et al. 2015).

The aetiology of chronic neck pain is complex with considerations encompassing the structural cause of the pain being the cervical facets, capsule, ligaments and/or musculature (Ita et al. 2017) as well as ergonomics, individual, behavioural and psychosocial factors (Genebra dos Santos et al. 2017). The complexities of chronic neck pain result in varying treatment strategies from conservative therapies to invasive procedures, such as cervical discectomy (Evans 2014). Conservative treatment consists of exercise therapies, medication, transcutaneous nerve stimulation and traction, with mobilisation and manipulation recommended as the first option (Bronfort et al. 2001; Evans 2014; Shaw, Shaw & Brown 2015; Van Eerd et al. 2010). In this regard, spinal manipulation may assist in the reduction of neck pain. Specifically, Cramer et al. (2006) have demonstrated that spinal manipulation, such as chiropractic manipulation, can reduce pain perception. Mechanisms by which manipulation may mediate neck pain relief may be because of its effect on the central nervous system (Schmidt et al. 2008). Schmidt et al. (2008) highlighted that this pain-mediating effect occurs at a supraspinal level and involves activation of the dorsal periaqueductal grey (dPAG) (Wright 1995), which, in turn, has a hypoalgesic effect resulting in pain inhibition (McCarthy 2010). In addition, joint motion fixations and restrictions, for which spinal manipulation is indicated, are thought to result in the formation of joint contractures and adhesions (Peterson & Bergmann 2002). It is purported that contractures and adhesions may cause involuntary changes in muscle excitability, resulting in pain (Katavich 1998; Peterson & Bergmann 2002). Manipulation has local effects on joints, joint capsules, ligaments and muscles, which result in reflex muscle relaxation as well as the breakdown of adhesions and thereby reduction of pain (Esposito & Philipson 2005; Gatterman 2005; Peterson & Bergmann 2002).

Recent findings suggest that daily ergonomics, such as standing, sitting and sleeping postures, need to be addressed as an adjunct management tool irrespective of the type of treatment that an individual is undergoing for neck pain relief (Canivet et al. 2008). Specifically, because an estimated one-third of an individual’s life is spent on sleeping, it has been proposed that there may be benefit in determining the best sleeping support system in treating neck pain (Erfanian, Tenzif & Guerriero 2004). The use of sleep support systems may be especially important in neck pain relief because humans have no active control of positioning and posture while sleeping (Leilnahari et al. 2011). This results in the spine being particularly vulnerable to abnormal mechanical forces, such as lateral bending while sleeping (Gordon, Grimmer & Trott 2007). As such, the use of a correct pillow may prove essential in neck pain relief. This is because it is purported that a pillow that functions to conform to the cervical spine lordosis and serves to support the head is ideal for preventing neck pain (Persson 2009). However, the converse is true in that an incorrect pillow may prevent the adaptation and sustained ‘end-range of motion postures’, resulting in stimulation of pain-sensitive structures and, consequently, neck pain (Gordon et al. 2007; Levangie & Norkin 2005). In this regard, Gordon, Grimmer and Trott (2009) conducted a study investigating the effect of five different pillow types on waking cervical pain, sleep quality and comfort. Gordon et al. (2009) found that feather pillows were consistently poor performers; polyester and foam pillows performed equally well to the participants’ own pillow and foam contour pillows were generally less comfortable and resulted in poorer quality sleep. Furthermore, water-based pillows were found to be more beneficial when treating cervical pain as compared to roll and standard pillows (Lavin, Pappagallo & Kuhlemeier 1997). Current studies investigating the best properties of a pillow indicate that pillows must be supportive so as to decrease the biomechanical stress on the cervical spine during sleep, must be of intermediate height and must be made of a material that does not result in excessive pressure (Gordon et al. 2007). However, the ultimate shape is still debatable with various studies showing conflicting results (Gordon et al. 2009; Lin & Wu 2015). According to Hager et al. (2001), visco-elastic polyurethane (VEP) foam is an ideal material for a pillow, as it has ‘conformable’ properties, and is able to mould to the individual’s neck shape (Jacobson et al. 2010). Furthermore, VEP foam also dampens sound and vibration, and absorbs shock and energy, all of which are important for quality sleep, and may assist in pain relief because sleep quality has been implicated in neck pain relief (Call-Schmidt & Richardson 2003; Edwards et al. 2008; Lautenbacher, Kundermann & Krieg 2006; Moldofsky 2001, 2008; Onen et al. 2001).

Chiropractic spinal manipulation has been shown to be beneficial in the treatment of chronic neck pain (Bronfort et al. 2001), as has the use of ergonomically sound cervical support pillows. A VEP pillow, despite its indicated advantages, has not specifically been investigated in conjunction with chiropractic manipulation in the treatment of this prevalent and debilitating condition.

### Aim of the study

The aim of this study was to determine if there is any additive effect to the inclusion of a VEP pillow with chiropractic treatment for chronic neck pain.

## Research method and design

### Design

This study used a 3-week quantitative pre- and post-test experimental design with random group allocation, whereby one group received chiropractic manipulation (CHI) and was compared with another group that received chiropractic manipulation combined with the use of a VEP foam cervical spine pillow (CHI+P).

### Sampling

**FIGURE 1:**
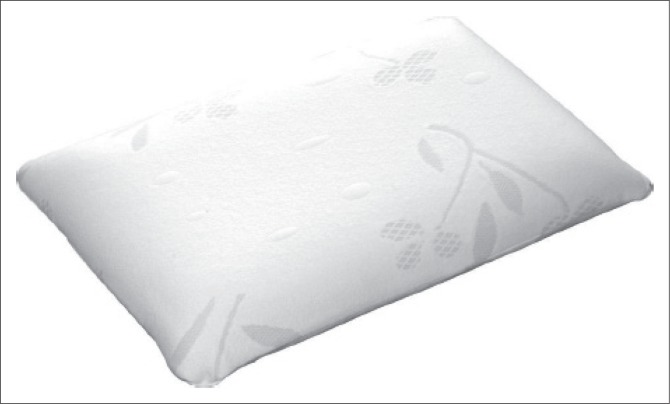
Visco-elastic polyurethane Memory Foam^®^ pillow (62 cm × 40 cm × 12 cm).

A total of 30 participants, both men and women, between the ages of 18 and 38 years, diagnosed with chronic neck pain who presented to the chiropractic clinic took part in this study (Table 1). The symptom of pain needed to be consistently present for more than 3 months before a diagnosis of chronic neck pain could be made. Participants were excluded if they were stomach sleepers or had any neurological symptoms associated with the neck pain. Participants were made aware of the study by flyers distributed in the chiropractic clinic and word of mouth. Recruitment occurred between January and February 2013. Participants were randomly allocated by drawing their prospective groupings from a sealed container (*n* = 15 per group). The participants in the CHI group were treated only with chiropractic manipulation performed by the researcher to the restricted segment/s of the cervical spine. The participants in the CHI+P group were treated with chiropractic manipulation to the restricted segment/s of the cervical spine as well as receiving a 62 cm × 40 cm × 12 cm VEP pillow after the first treatment consultation (Figure 1).

**TABLE 1:** Demographic and baseline characteristics of participants in each group.

Variable	Chiropractic treatment only group (CHI) (*n* = 15)	Chiropractic treatment with visco-elastic polyurethane foam cervical spine pillow group (CHI+P) (*n* = 15)
Age distribution (years)	22–30	22–38
Mean age (years)	25.13 ± 2.13	26.07 ± 4.30
Females (*n*)	7	8
Males (*n*)	8	7

Note: Data are presented as mean ± SD.

### Data collection

Subjective, quantitative data were collected during the trial between February and May 2013 by means of the Numerical Pain Rating Scale (NRS) (Farrar et al. 2010) and the Vernon–Mior Neck Pain and Disability Index (NDI) (Vernon 1996). Measurements were recorded by the researcher at the first and fourth consultations prior to treatment. After completion of the six treatments over the 3-week period, there was a final seventh consultation where no treatment occurred, but only final measurements conducted. The NRS is a standard questionnaire used in chronic pain studies and has proven valid and reliable in a variety of settings (Farrar et al. 2010, 2008). Each participant was required to mark 1 of 11 boxes rated from 0 to 10, where 0 indicated no pain and 10 represented the ‘worst imaginable pain’ (Marquie et al. 2008). In addition to statistical significance, the present study attempted to determine clinical significance. In this regard, according to Salaffi et al. (2004), a minimum clinically important difference (MCID) of 2 points (33%) is an indication that the participant is feeling ‘much better’.

In addition, the present study utilised the NDI, which is a valid and reliable (Ackelman & Lindgren 2002) revised version of the Oswestry Index, which measures the impact and effect of neck pain on the day-to-day life of patients (Vernon 1996). Using this questionnaire, each participant was required to complete the questionnaire by indicating which statement best suited and described their condition using a rating of 0–5, with 0 indicating the least impact and 5 the highest. The NDI has 10 sections to be completed with a possible maximum score of 50 points. An advantage of using the NDI scoring system is that the MCID can also be determined. Specifically, Young et al. (2009) indicated that an MCID in the NDI of 7.5 points equates to a 15% improvement in neck pain and the patient’s day-to-day life.

### Treatments

Participants in both groups underwent a total of six chiropractic treatments performed by the researcher over a 3-week period between February and May 2013. Chiropractic treatments consisted of chiropractic manipulation of the cervical spine using a diversified technique (Esposito & Phillipson 2005). This technique is the form of high-velocity, low-amplitude thrust that is traditionally associated with chiropractic manual adjustments. For this method, a short (low-amplitude), quick (high-velocity) thrust was delivered over the restricted joints (one at a time up to a maximum of three) with the goal of restoring normal range of motion in the joint. Each participant’s body was positioned in specific ways to optimise the adjustment of the spine (Esposito & Phillipson 2005). The restrictions to be manipulated were identified by means of motion palpation of the cervical spine. If more than three restrictions were found during motion palpation, the three with the highest degree of restriction were treated. Chiropractic treatments took place for both the CHI and CHI+P participants at the Chiropractic Day Clinic in Gauteng, South Africa. In addition to the chiropractic treatments, participants in the CHI+P were provided with a 62 cm × 40 cm × 12 cm VEP pillow (Memory Foam^®^, Sleep Active, South Africa) and with instructions on the pillow’s use. The instructions explained that the whole head and face should be cradled by the pillow by placing the head in the middle of the pillow. It was advised to side or back sleep with the pillow, depending on participant preference. Compliance of pillow use was verbally confirmed and documented at each consultation by the researcher.

### Data analysis

Data analysis was performed by an independent statistician using raw scores provided by the researcher. Normality of distribution was determined for all variables using the Shapiro–Wilk test. Non-parametric tests were utilised to determine the effects of chiropractic treatments compared to chiropractic treatment combined with a VEP pillow on the patient’s perception of neck pain. The Friedman test was used to determine if a change occurred from pre- to post-test within each group, while the Wilcoxon signed-rank test was utilised to determine if a change took place within each group (i.e. visit 1 to visit 4 or from visit 4 to visit 7). The Mann–Whitney *U* test was used to determine if any intergroup differences existed between the two groups. Statistical significance was set at *p* ≤ 0.05 and data were analysed by the Statistical Consultation Services (STATKON) of the University of Johannesburg using commercial software (SPSS version 21, Chicago, IL). Data are displayed as means ± standard deviation (SD).

### Ethical consideration

The protocol was designed according to the ethical norms set out in the 1961 Helsinki Declaration (modified in Edinburgh in 2000) and the study was approved by the university’s Research Ethics Committee. Written informed consent was obtained from the participants after they were explained the purpose of the study, measurement procedures and the possible negative events that could be encountered during the study. University of Johannesburg Research Ethics Committee; AEC 13-01-2013; 4 March 2013.

## Results

### Numerical Pain Rating Scale

**FIGURE 2:**
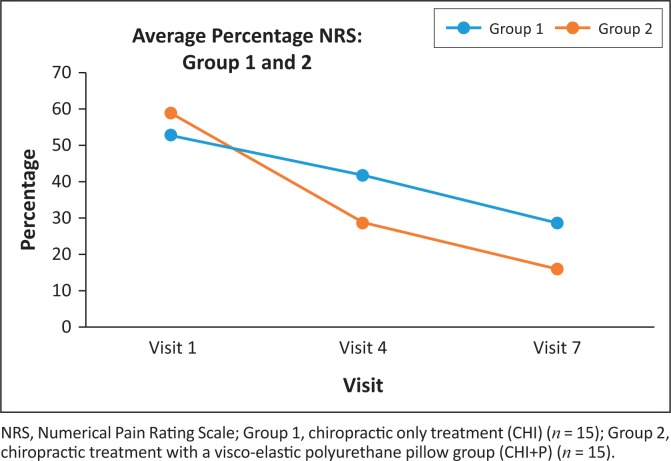
Numerical Pain Rating Scale percentage changes following the introduction of a visco-elastic polyurethane foam pillow and/or chiropractic treatment protocol in patients with chronic neck pain caused by cervical facet syndrome.

The Mann–Whitney *U* test demonstrated that the two groups were homogenous at baseline (*p* = 0.41). Both the CHI and CHI+P were found to have statistically significant (*p* ≤ 0.05) improvements in their NRS scores from consultations 1 to 4 (*p* = 0.016 and *p* = 0.001, respectively) and from consultations 4 to 7 (*p* = 0.002 and *p* = 0.009, respectively). Similarly, both the CHI (*p* = 0.001) and the CHI+P (*p* = 0.001) demonstrated significant improvements in their NRS scores from pre- to post-test (consultations 1–7). The groups were heterogeneous at consultations 4 (*p* = 0.041) and 7 (*p* = 0.015). Figure 2 demonstrates the percentage change in NRS between the two groups. According to Salaffi et al. (2004), a change of 2 points (33%) in the NRS indicates that the participant is feeling ‘much better’. As a mean, both groups achieved more than a 33% improvement in the NRS following the respective treatment interventions. However, the CHI+P’s NRS scores were substantially higher at 73% compared to the CHI’s 43% increase (Table 2).

**TABLE 2:** Changes in chronic neck pain caused by cervical facet syndrome following the introduction of a visco-elastic polyurethane foam pillow and/or chiropractic treatment protocol.

Variable	Chiropractic treatment only group (CHI) (*n* = 15)			Chiropractic treatment with visco-elastic polyurethane foam cervical spine pillow group (CHI+P) (*n*=15)		
	Consultation 1	Consultation 4	Consultation 7	Consultation 1	Consultation 4	Consultation 7
NRS score	5.27 ± 1.94	4.27 ± 1.58†	2.87 ± 1.19‡,§	5.93 ± 1.22	2.93 ± 1.75†	1.60 ± 1.30‡,§
NDI score	10.93 ± 5.50	6.33 ± 2.58†	4.20 ± 2.18‡,§	13.40 ± 4.82	6.40 ± 5.33†	3.80 ± 4.28‡,§

Data are presented as mean ± SD.

† , Consultation 4 statistically significant (CHI: *p* = 0.016 [NRS]; *p* = 0.001 [NDI]; CHI+P: *p* = 0.001 [NRS]; *p* = 0.001 [NDI]) compared to consultation 1.

‡ , Consultation 7 statistically significant (CHI: *p* = 0.02 [NRS]; *p* = 0.02 [NDI]; CHI+P: *p* = 0.009 [NRS]; *p* = 0.001 [NDI]) compared to consultation 4.

§ , Consultation 7 statistically significant (CHI: *p* = 0.001 [NRS]; *p* = 001 [NDI]; CHI+P: *p* = 0.001 [NRS]; *p* = 0.003 [NDI]) compared to consultation 1.

NRS, Numerical Pain Rating Scale; NDI, Vernon–Mior Neck Pain and Disability Index.

### Vernon–Mior Neck Pain and Disability Index

**FIGURE 3:**
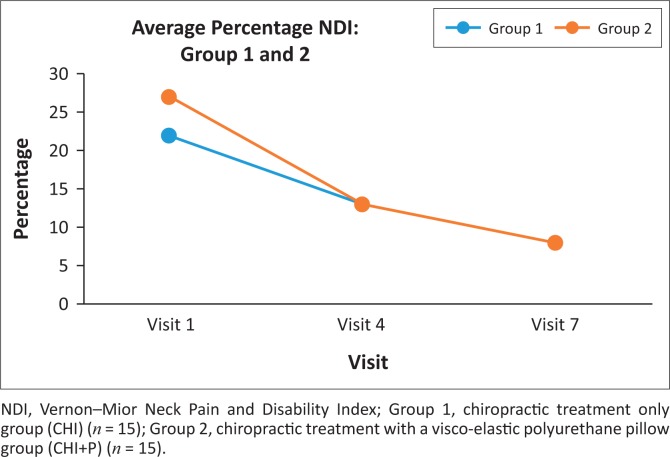
Vernon–Mior Neck Pain and Disability Index percentage changes following the introduction of a visco-elastic polyurethane foam pillow and/or chiropractic treatment protocol in patients with chronic neck pain caused by cervical facet syndrome.

Both the CHI and CHI+P were found to be homogenous at the commencement of the study (*p* = 0183). Statistical evaluation of the NDI of the CHI and CHI+P demonstrated statistically significant improvements from consultations 1 to 4 (*p* = 0.001 for both groups), 4 to 7 (*p* = 0.002 and *p* = 0.003, respectively), as well as from consultations 1 to 7 (*p* = 0.001 for both groups). In addition, the Mann–Whitney *U* test demonstrated that the groups remained homogenous at consultations 4 (*p* = 0.574) and 7 (*p* = 0.195). Figure 3 demonstrates the percentage change in NDI scores for each group at consultations 1, 4 and 7. When scrutinising the overall percentage improvement of both groups, the CHI demonstrated a 59% improvement, while CHI+P reported a 71% improvement. According to Young et al. (2009), a score change of 7.5 (or 15%) represents a minimal clinically significant improvement in the NDI.

## Discussion

The purpose of this study was to determine if the inclusion of a VEP pillow could benefit the chiropractic treatment of chronic neck pain. In this regard, the present study demonstrated that both the CHI and CHI+P groups improved their mean NRS and NDI scores. These findings are not unusual in that it has previously been demonstrated that chiropractic manipulation is effective in the treatment of chronic neck pain (Bryans et al. 2014) because of the manipulation mechanical and/or neurological effects (Gatterman 2005; Katavich 1998; Peterson & Bergmann 2002). However, a novel finding was that the addition of the VEP pillow resulted in an improvement in an MCID. Specifically, the CHI+P demonstrated a clinical improvement in NRS scores of 73% compared to the CHI’s 43%. Similarly, the CHI+P demonstrated a 71% in their NDI scores compared to the CHI’s 59%.

While the benefits of cervical pillows on reducing neck pain are well documented (Erfanian et al. 2004; Persson 2009; Persson & Moritz 1998), a novel finding of the present study is that the addition of a VEP to chiropractic treatment may provide additional improvements in neck pain beyond the pain-mediating effect (Schmidt et al. 2008). This additional benefit may have arisen because the pillow may have added support to the cervical spine during sleep, thus improving or even correcting poor sleeping posture in this study (Persson 2009). The facet joints are a common cause of chronic neck pain (Ita et al. 2017). Moreover, it has been shown that biomechanical loading of the facet joint capsule can lead to pain (Ita et al. 2017). By addressing sleep ergonomics during the time of receiving chiropractic manipulation, it may be hypothesised that the facet joints may have had an improved chance to repair an injury. In this regard, chiropractic manipulation targets facet joints and restores abnormal kinematics allowing for improved nutrition and hydration to the joint. The simultaneous use of an ergonomically sound pillow may have kept the joint in an open pack position, further improving tissue repair during sleep (Persson 2009). Alternatively, the addition of the pillow in this study may have improved sleep quality and improved waking pain (Lin & Wu 2015). It should also be noted that improved sleep quality not only decreases waking pain, in itself, but also results in an improved secretion of growth hormone during sleep, which further facilitates tissue repair (Lange et al. 2006; Van Liempt et al. 2001).

While the concomitant use of a pillow with chiropractic treatment is a novel finding, many studies exist that attempt to improve the efficacy of chiropractic manipulation with the addition of additional therapies in a multimodal form of therapy (Bryans et al. 2014; Gross, Hoving & Haines 2004; Hurwitz 2008). Such additional therapies include inter alia advice or education, stretching, exercise and pulsed short-wave therapy. However, it must be noted that the addition of other therapies to chiropractic treatment does not always result in a benefit, but may indeed cause an interference effect and actually reduce the efficacy of the chiropractic treatment itself (Bryans et al. 2014). This was, however, not the case in the present study.

## Limitations

Some limitations should be noted in this study that may have had an influence on the results. Chance cannot be excluded because of the small sample size. The small sample does allow for some insights, but no definitive conclusions can be made. Potential researcher bias should also be considered as an influence in the results, ideally researcher blinding should have occurred for treatment groups as well as measurements. Also, tighter external variables could have been controlled for, such as occupation and age of the participants. The low mean age of the participant sample may have made them more receptive to treatment than the average population suffering with chronic neck pain.

## Recommendations

While the findings of the present study are novel and have important implications for clinical treatment and therapeutic guidelines, future studies should make use of additional objective measures, such as surface electromyography (sEMG) of cervical musculature, pressure algometer readings of the cervical facets and cervical range of motions, to aid the clinical findings. In addition, future studies should be of a longer duration to maximise benefits because individuals take time to adapt to newly introduced pillows and sleeping posture. In this regard, Gordon et al. (2009) propose the use of a 1-week ‘washout period’ which may allow participants time to adapt. Future studies should also include a larger sample size, blinding of the treatment and measurements, crossover design and/or a follow-up at 3 and 6 months. It would also be beneficial to compare various types of pillows on neck pain in addition to chiropractic treatment to determine the optimal multimodal treatment for neck pain.

## Conclusion

Combining the mechanical and pain inhibitory effects of cervical manipulation with the supportive properties of the VEP foam pillows may be beneficial to the patient compared to chiropractic treatment alone. While both the chiropractic treatment and the combined treatment proved effective, the clinical performance of the CHI+P group with regard to NRS indicates a potential synergistic effect of the VEP foam pillow with cervical manipulation in the treatment of chronic neck pain. There were trends in the clinical analysis of the NDI, which also indicated some additional benefit. Further research is warranted.
